# Disordered eating and internalizing symptoms in preadolescence

**DOI:** 10.1002/brb3.1904

**Published:** 2020-10-19

**Authors:** Kai S. Thomas, Marc O. Williams, Ross E. Vanderwert

**Affiliations:** ^1^ School of Psychology Cardiff University Cardiff UK; ^2^ Cardiff University Centre for Human Developmental Science Cardiff University Cardiff UK

**Keywords:** anxiety, depression, disordered eating, eating disorders, preadolescence

## Abstract

**Objectives:**

Research has demonstrated links between disordered eating, anxiety, and depression in adults and adolescents but there is limited research investigating these associations in preadolescence. The current study examined the associations between disordered eating, anxiety, and depression during preadolescence, as well as the role of gender in moderating these associations.

**Method:**

Two hundred and thirteen children (*M* = 10.3 years; 51.2% male) reported levels of disordered eating (ChEAT) and anxiety and depression symptoms (RCADS‐25).

**Results:**

Regression analyses support an association between disordered eating and both anxiety and depression in preadolescence. Overall, there were no significant differences between boys and girls when the main effect was examined, which differs from research in adolescents.

**Discussion:**

These findings highlight the importance of early detection for disordered eating behaviors and attitudes, as well as anxiety and depression in both boys and girls during preadolescence. Longitudinal research examining these associations is vital to help understand the trajectories of these problems, but also the gender differences in disordered eating that emerge during adolescence. Transdiagnostic interventions targeting several co‐occurring problems, such as disordered eating, anxiety, and depression might be effective for preventing the development of eating disorders in the long term.

## Graphical abstract

This study examined the associations between disordered eating, anxiety, and depression in 213 preadolescents using self‐report questionnaires. There were strong associations between anxiety, depression, and disordered eating, and no gender differences present. These findings highlight the importance of early detection for disordered eating behaviors and attitudes, as well as anxiety and depression in both boys and girls during preadolescence.

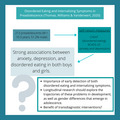

## 1 INTRODUCTION

Research on the etiology and prevalence of diagnosed eating disorders, such as anorexia nervosa and bulimia nervosa, and disordered eating has mainly focused on adolescents and adults (e.g., Kaye, 2008; Naor‐Ziv & Glicksohn, 2016; Schmidt & Treasure, 2006). Eating disorders are serious mental illnesses that typically develop during or after puberty; therefore, research has focused on adolescents as a population at increased vulnerability (Parkinson et al., 2012). Indeed, a UK‐based longitudinal study reported prevalence rates of 0 at 5 years, increasing to 3.56 in 100,000 at 11–12 years and nearly tripling to 9.51 in 100,000 by 12–13 years (Nicholls et al., 2011), highlighting the need for research to understand the etiology of eating disorders in early childhood.

Disordered eating refers to the behaviors and attitudes that are not severe or frequent enough to meet criteria for an eating disorder diagnosis (Graber et al., 1994). These behaviors can include calorie counting, restricting food intake, over‐exercising, binge eating, and avoidance of certain food types; have been found to emerge in childhood (Killen et al., 1994; Leon et al., 1993); and increase the risk of developing diagnosable eating disorders in adolescence (Evans et al., 2017). The strongest predictor of disordered eating behaviors later in adolescence is the degree of disordered eating already present in early adolescence (Attie & Brooks‐Gunn, 1989; Wichstrøm, 2000), which suggests that disordered eating, once present, tends not to resolve spontaneously and can intensify. A comprehensive review by Culbert et al. (2015) highlights a clear need for longitudinal research examining high‐risk samples with elevated disordered eating, advising studies start in preadolescence to capture risk trajectories and antecedents of eating pathology. Particular factors of interest in the development of disordered eating, and in turn eating disorders, are internalizing disorders such as depression and anxiety, which have high comorbidity with eating disorders (Godart et al., 2007). Pallister and Waller (2008) propose a model of shared transmission to account for the comorbidity between anxiety and eating disorders in which they share etiological factors such as harm avoidance cognitions and safety behaviors. Keel et al. (2005) examined this model by comparing monozygotic (MZ) twin pairs discordant for eating disorders and anxiety disorders. Within MZ twin pairs discordant for eating disorders, twins who did not have an eating disorder were at increased risk for anxiety disorders compared with controls. Similarly, for unaffected twins within MZ pairs discordant for anxiety disorders, there was an increased risk for eating disorders compared with controls (Keel et al., 2005). Studies exploring the trajectories of anxiety and eating disorders are mostly retrospective and have found that adolescents and adults with an eating disorder frequently report a childhood‐onset anxiety disorder, with an average onset at 8–10 years (Adambegan et al., 2012; Kaye et al., 2004; Raney et al., 2008).

Studies have also examined trajectories of disordered eating behaviors and internalizing symptoms in community samples of adolescents. For example, Puccio et al. (2017) assessed longitudinal bidirectional effects of disordered eating, anxiety, and depression, from age 15 to 18.5 years of age. They found for both girls and boys, disordered eating, specifically eating and shape/weight concerns, was a risk factor for anxiety; whereas depression was a risk factor for eating concerns. In addition, body dissatisfaction in both male and female adolescents has also been found to predict depression symptoms across time (Ferreiro et al., 2014). Body dissatisfaction has been found to be strongly associated with disordered eating (Fig ueiredo et al., 2019), with some measures of disordered eating specifically incorporating examinations of body dissatisfaction, for example, the Children's Eating Attitude Test (ChEAT; Maloney et al., 1989) has items that focus on body shape and weight. This raises some concerns around the predictive value of body dissatisfaction independent of general disordered eating behaviors. Guided by Pallister and Waller’s (2008) model above, this study will examine general internalizing symptoms that would be more indicative of general cognitive and affective tendencies predisposing one to disordered eating, including body dissatisfaction.

To date, there is a shortage of research examining the factors associated with disordered eating in preadolescent children in primary school. One such study by Holm‐Denoma et al. (2014) explored the relations between internalizing symptoms and disordered eating in a sample ranging from 7 to 16 years. They reported similar levels of disordered eating present in both boys and girls between ages 9 and 11, but disordered eating levels were higher for girls than boys at age 15. The authors suggest the gender differences in levels of disordered eating emerged between ages 12 and 15, with a linear increase in disordered eating observed in girls and stable levels among boys (Holm‐Denoma et al., 2014). Associations between disordered eating and depression were already present by 10 years in both boys and girls. This association increased between ages 9 and 11 in boys and then remained stable between ages 12 and 15, whereas in girls an opposite pattern was observed, in which the association between disordered eating and depression remained stable between ages 9 and 11 and was followed by an increase in the association between ages 12 and 15. A weak and largely nonsignificant association was found between anxiety symptoms and disordered eating across age cohorts and gender, which is in contrast to comorbidity reported in previous studies between anxiety disorders and disordered eating in older adolescents, especially females (e.g., Touchette et al., 2011; Zaider et al., 2000), and is at odds with the finding that childhood anxiety disorders often precede the development of eating disorders (Godart et al., 2000). Importantly, the measures used to examine disordered eating in the study by Holm‐Denoma et al. (2014) were not adapted for the youngest ages in their sample. Further research utilizing validated measures in preadolescents is needed to address these inconsistent findings.

In contrast to the nonsignificant associations between anxiety and disordered eating reported by Holm‐Denoma et al. (2014), research by Houldcroft et al. (2014) in preadolescent children (mean age 8.8 years) found that dietary restraint and emotional eating were positively associated with reported general and social anxiety, as well as depression symptoms. In addition, significant gender differences were found in reports of general and social anxiety, with a higher number of symptoms reported by girls than boys (Houldcroft et al., 2014). The study aimed to measure eating behaviors through an examination of dietary restraint, emotional eating, and external eating; however, these measures are more focused on behaviors present in bulimia nervosa and binge eating (e.g., Johnson et al., 2012; Stein et al., 2007), rather than eating disorder symptoms more broadly. Ferreiro et al. (2012) examined gender differences in depression, disordered eating and the co‐occurrence of these from preadolescence to mid‐adolescence. They reported gender differences emerge around age 12 for disordered eating, and age 14 for co‐occurring disordered eating and depression. These authors did not measure anxiety alongside depression, preventing the examination of any differential association of these presentations with disordered eating and gender. Another limitation of measuring depression in isolation is that its comorbidity with anxiety (Angold & Costello, 1993) might lead to a spurious association with disordered eating that would be better explained by anxiety.

Given the paucity of research in preadolescents that has investigated both anxiety and depression and used measures of disordered eating validated for preadolescence, this study aims to build upon the literature to explore concurrent associations between disordered eating and internalizing symptoms using a validated measure for preadolescents. This will allow for addressing inconsistencies in the existing literature with respect to the association of internalizing symptoms with disordered eating, and by including a demographically diverse sample of boys and girls, will allow for gender differences in these associations to be examined. We hypothesize that children who report higher levels of disordered eating behaviors and attitudes will also report higher levels of anxiety and depression symptoms, and that there will be higher levels of anxiety and depression symptoms among children who are categorized as above the clinical threshold for disordered eating, than for children who are categorized as below clinical threshold. It is also hypothesized that anxiety and depression will both be significant independent variables in regression models examining relations between anxiety, depression, and disordered eating. We further aim to examine the role gender may play in moderating the relations between disordered eating, anxiety, and depression and investigate whether gender plays a role in how this symptomatology presents in preadolescence. In line with previous research in nonclinical preadolescent children, we hypothesize that there will be no gender differences in disordered eating or depression symptoms. Based on literature from adolescents, we hypothesize that gender will moderate the association between anxiety symptomatology and disordered eating, with stronger associations between disordered eating and anxiety present in girls compared to boys.

## 2 METHODS

### 2.1 Ethical guidelines

This project received approval from the Cardiff University School of Psychology Ethics Committee (EC.19.02.12.5566GR3A3). Opt‐in parental consent was obtained as well as assent from the child prior to participating in the study. Both parent and child were provided with a description of the study and were made aware of their right to withdraw at any stage of the study. All data were stored anonymously.

### 2.2 Recruitment and sample

The study recruited a sample of 213 children aged 9–11 years (*M* = 10.3 years; 51.2% male) from twelve state‐run primary schools in south Wales. An additional 18 children with parental consent to participate in the study were absent on the day of testing.

**Figure 1:**
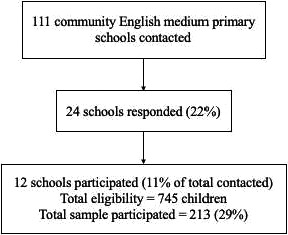
Recruitment process for schools

Head teachers from all 111 community English‐medium primary schools in south Wales were contacted to enquire about interest in the study. Children were recruited from both years 5 and 6. Twenty‐four schools responded to the invitation (22%), but only twelve were able to participate in the research (11% of total schools contacted). Barriers to participation for some of these schools included no current year 5/6 classes and lack of time due to commitments to other projects. See Figure 1 below for a summary of the recruitment process.

G*Power 3.1 (Faul et al., 2007) was used to conduct a power calculation to determine the required sample size for correlation and multiple regression analyses. Based on previous literature, a medium effect size of 0.18 measured by Cohen's f^2^ was used as well as an alpha value of 0.05, and a maximum of three independent variables. The estimated total sample size was 100 participants.

Free school meal (FSM) uptake data was collected for each school in order to assess socioeconomic status at the school level. This figure is a three‐year average of the pupils eligible for FSM and the Welsh average is 18% (Welsh Government, 2019). High FSM averages indicate high material deprivation; however, this is only at the school level and not at the individual family level. Table 1 below provides a summary of the participation rates for each school, as well as FSM data, proportion of children with reported additional learning needs (ALN) or special education needs (SEN), and the proportion of children who are learning English as an additional language (EAL). The data were collated from a Welsh Government school information database (Welsh Government, n.d.).

**Table 1:** Individual school characteristics: participation rates and proportion of children in each school who meet criteria for FSM, ALN/SEN, and EAL provision

	*N* (proportion of eligible children)	FSM (%)	ALN/SEN (%)	EAL (%)
School 1	46 (38%)	24.5	22.3	8.4
School 2	10 (20%)	50.4	33.7	16.3
School 3	28 (27%)	13.0	14.9	N/A
School 4	35 (37%)	16.8	27.6	16.1
School 5	17 (34%)	40.3	34.8	3.8
School 6	10 (14%)	40.5	35.8	25.5
School 7	2 (9%)	45.1	30.1	15.7
School 8	21 (35%)	2.0	5.9	1.3
School 9	5 (17%)	13.0	18.4	N/A
School 10	8 (27%)	27	22.4	8.8
School 11	11 (22%)	30.4	17.9	2.9
School 12	20 (33%)	7.2	9.7	6.3

Abbreviation: ALN, Additional Learning Needs; EAL, English as an Additional Language; FSM, Free School Meals; SEN, Special Educational Needs.

### 2.3 Measures

#### 2.3.1 Children's Eating Attitude Test

Disordered eating behaviors and attitudes were measured using the Children's Eating Attitude Test (ChEAT), a self‐report modified version of the abbreviated adult Eating Attitudes Test (EAT‐26; Garner & Garfinkel, 1979; Maloney et al., 1989). The ChEAT is a 26‐item questionnaire designed to measure dimensional disordered eating behaviors and attitudes in children aged between 8 and 13 years old, such as food preoccupation, concerns about being overweight, bingeing and purging, and dieting. Each item is rated by the child using a 6‐point response format (always, very often, often, sometimes, rarely, never) and represents the frequency with which the child demonstrates the attitude or behavior described in each item (e.g., I feel very guilty after eating.). Scores range from 0 to 78, with the three most symptomatic responses (“often,” “very often” and “always”) scored from 1 to 3, respectively, and the remaining three responses scored as zero.

The scale has good test–retest reliability (α = 0.81) and good internal consistency (α = 0.9) in boys and girls aged between 7 and 12 years (Maloney et al., 1988; Smolak & Levine, 1993). Higher total scores on the ChEAT indicate higher levels of symptomatology, with a cutoff score of 20 indicative of more severe eating pathology that could warrant further clinical assessment (Maloney et al., 1988).

Adjustments to the wording of item 4 were made where “I have gone on eating binges where I feel that I might not be able to stop” was changed to “I have started to eat and then felt like I cannot stop.” This was based on previous studies (e.g., Coombs et al., 2011) highlighting difficulties with the comprehension of “binges” with children of a similar age. In addition, items 9 and 26, which refer to “vomit,” were also accompanied by “am/be sick,” a more familiar term for children. Finally, item 21 was changed from “I give too much time and thought to food” to “I spend too much time thinking about food,” in order to simplify the wording and improve comprehension. Alpha values for the adjusted items were acceptable (α = 0.706).

#### 2.3.2 Revised Child Anxiety and Depression Scale ‐ 25 item version

The revised Child Anxiety Depression Scale (25‐item version; RCADS‐25; Muris et al., 2002) is a brief assessment of anxiety and depression symptoms as defined by the DSM. The anxiety and depression subscales are comprised of 15 and 10 items, respectively. Cronbach's alpha values for the current sample were acceptable for both scales (anxiety: α = 0.864; depression: α = 0.839), as well as the total score (α = 0.916). All 25‐items are rated on a 4‐point scale (never, sometimes, often, always) and represent the frequency to which these behaviors, thoughts, or feelings occur (e.g., I have trouble sleeping). Overall scores range from 0 to 75 and individual responses are scored from 0 (never) to 3 (always). Higher scores indicate more severe anxiety and depression symptomatology. The RCADS‐25 is comparable to the full‐length version regarding test–retest reliability (*r*s = .78–.86, *p* < .001) and internal consistency (α = 0.87–0.95; Brown et al., 2014).

### 2.4 Procedure

Head teachers provided consent for the study to take place at the school and opt‐in consent was required from parents/guardians. All questionnaires were administered within schools during class time. The children whose parents/guardians provided consent were assessed in groups of 5–6 at a time, where they were seated in a separate classroom with the researcher who was either alone or accompanied by an additional teacher. Children were provided with an age‐appropriate introduction to the study and the researcher, as well as an opportunity to ask questions. Children were asked to not share their answers with anyone else or read each other's answers.

A verbal debrief was provided at the end of the session to answer any questions the children had and a written debrief was sent home to provide a list of contact details for the researcher and their supervisor, as well as some support organizations for advice and helpful resources. Children were observed during the testing session for signs of emotional distress and in the event of this occurring, the child was encouraged to discontinue their participation (*n* = 0). The questionnaire measures used in this study were not diagnostic tools, so diagnoses could not be determined based on the children's scores. In the event of an extremely high score on these measures, a protocol was put in place to inform the parent and advise a visit to their medical doctor for further support (*n* = 0).

### 2.5 Data analysis

All statistical analyses were performed using SPSS version 25.0 (IBM Corp., 2017). All analyses were two‐tailed and a *p*‐value of .05 was used to determine statistical significance.

Data were screened for floor and ceiling effects, as well as missing values. There were 33 data points missing from the ChEAT data (0.6% of overall data points) and 34 data points missing from the RCADS data (0.6% of overall data points). These missing values were distributed across the items in these scales. Visual inspection of plots revealed that the data were positively skewed and not normally distributed; this was supported by Shapiro–Wilk normality tests which were significant for both ChEAT score and all RCADS measures.

Therefore, a log transformation was performed on both RCADS and ChEAT measures. All measures were found to be normally distributed after the log transformation and could therefore be analyzed with parametric tests. In light of some limitations raised regarding the use of log transformations (Field & Wilcox, 2017), bootstrapping using the original data was also performed alongside each statistical test to ensure the results were robust. Results using parametric tests and bootstrapping were identical unless reported.

Correlational analyses were used to explore associations between disordered eating, anxiety and depression. The significant associations were followed up with linear regressions, whereby ChEAT scores were entered into the model as the dependent variable and anxiety and depression symptoms were independent variables. In order to examine whether gender may play a moderating role in the relations between anxiety, depression, and disordered eating, *t* tests were conducted to test for differences between boys and girls on both ChEAT and RCADS measures. Separate correlation and regression analyses were performed to examine associations between the questionnaire measures for girls and boys. In each of the linear regression models, ChEAT was entered as a dependent variable and anxiety and depression symptoms were independent variables. An additional linear regression was performed to examine the interaction between anxiety and gender when ChEAT was the dependent variable.

## 3 RESULTS

### 3.1 Descriptive statistics

Table 2 presents the descriptive statistics for the whole sample as well as split by gender. For the whole sample, ChEAT scores and RCADS total scores presented some variation in the sample, with increased anxiety compared to depression scores.

**Table 2:** Descriptive statistics for the whole sample and split by gender

	Whole sample (*n* = 213)		Male (*n* = 109)		Female (*n* = 104)		*t*(211)	*p*	*d*
	*M* (*SD*)	Range	*M* (*SD*)	Range	*M* (*SD*)	Range			
ChEAT	9.18 (6.85)	0–43	9.43 (6.71)	0–31	8.91 (7.01)	0–43	−0.558	.578	0.077
RCADS Total	20.73 (12.50)	1–68	20.03 (11.18)	2–55	21.46 (13.77)	1–68	−0.518	.605	0.071
RCADS Anxiety	12.74 (7.89)	0–38	12.11 (7.15)	1–29	13.39 (8.58)	0–38	−0.957	.340	0.033
RCADS Depression	7.99 (5.47)	0–30	7.92 (5.13)	0–26	8.07 (5.82)	0–30	−0.101	.919	0.014

Abbreviations: ChEAT, Children's Eating Attitude Test; RCADS, Revised Child Anxiety and Depression Scale.

^a^ Untransformed data are presented in the table.

### 3.2 Associations between disordered eating, anxiety, and depression symptoms

To test our first hypothesis, correlations were performed to investigate the associations between disordered eating, anxiety, and depression in the sample (Table 3). There was a significant positive correlation between ChEAT scores and total RCADS scores, as well as ChEAT scores and both anxiety and depression subscales.

**Table 3:** Pearson's correlations between questionnaire measures

	ChEAT	RCADS Total	RCADS Anxiety	RCADS Depression
ChEAT	1			
RCADS Total	0.421*	1		
RCADS Anxiety	0.417*	0.932*	1	
RCADS Depression	0.356*	0.880*	0.679*	1

Abbreviations: ChEAT, Children's Eating Attitude Test; RCADS, Revised Child Anxiety and Depression Scale.

Transformed data were used in the analyses.

* Correlation is statistically significant at *p* < .001

To further investigate the significant associations between disordered eating, anxiety, and depression, a linear regression was performed with RCADS total scores and ChEAT scores. The model was significant, *F* (1, 211) = 45.45, *p* < .001, *R* ^2^ = .177. Another linear regression was performed to examine the independent associations of the RCADS anxiety and depression subscales on ChEAT scores. The model was significant, *F* (2, 210) = 23.58, *p* < .001, *R* ^2^ = .183. While anxiety contributed significantly to the model (*B* = 0.357, *SE* *B* = 0.093, *p* < .001), depression was not significant (*B* = 0.144, *SE* *B* = 0.091, *p* = .113). As anxiety and depression often co‐occur and were correlated, collinearity diagnostics were examined to ensure assumptions of multicollinearity were not violated. VIF values were acceptable (VIF = 1.853).

### 3.3 Examination of gender differences

To test our hypothesis that there will be no gender differences present in disordered eating or depression, a *t* test was conducted to examine whether there were significant gender differences in average scores on the ChEAT and RCADS (Table 2). No significant differences were found for any of the mean scores between boys and girls.

Additional correlation analyses were performed to examine associations between the questionnaire measures for girls and boys, respectively (see Supporting information for results tables). Overall, the correlations were very similar for both genders. The correlations between ChEAT scores and total RCADS scores appear slightly stronger in girls (*r* = .442, *p* < .001) compared to boys (*r* = .404, *p* < .001). This is also the case for correlations between ChEAT scores and anxiety (girls: *r* = .480, *p* < .001; boys: *r* = .363, *p* < .001) and depression (girls: *r* = .378, *p* < .001; boys: *r* = .336, *p* < .001) subscales.

To test whether the RCADS anxiety coefficient was significantly different between boys and girls, a linear regression was performed with the RCADS anxiety term, a recoded dummy variable for gender, and an interaction term combining these two variables. The overall model was significant, *F* (3, 209) = 15.37, *p* < .001, *R* ^2^ = .181, however the interaction term was not a significant coefficient (*B* = 0.118, *SE* *B* = 0.138, *p* = .394), indicating that the RCADS anxiety coefficient did not differ significantly between boys and girls. The linear regression was repeated to test whether the RCADS depression coefficient differed significantly between boys and girls. The overall model was significant, *F* (3, 209) = 10.30, *p* < .001, *R* ^2^ = .129, however the interaction term was not a significant coefficient (*B* = 0.055, *SE* *B* = 0.138, *p* = .689), indicating that the RCADS depression coefficient did not differ significantly between boys and girls.

## 4 DISCUSSION

Research conducted with adolescents and adults has reported strong associations between internalizing symptoms and disordered eating behaviors, as well as diagnosed eating disorders; however, studies examining these associations in preadolescence have provided mixed results. The results of the current analyses provide strong support for a pattern of associations between disordered eating and internalizing symptoms in preadolescence that is consistent with what has been reported in adolescents and adults. Specifically, results showed that preadolescent children who reported higher levels of disordered eating also reported higher levels of anxiety and depression symptoms.

Consistent with our hypotheses, the relation between anxiety and disordered eating was statistically significant, but depression was not significant in the model. The relation between depression and disordered eating has previously been reported in preadolescents (e.g., Evans et al., 2017; Gardner et al., 2000); however, not all of these studies included anxiety as an independent variable in the model. The overlap between anxiety and depression could mean that once the variance in disordered eating is accounted for by anxiety, the unique variance explained by depression is no longer statistically significant. To our knowledge this is one of the first studies to highlight this dissociation between anxiety and depression using validated measures for preadolescents. Previous studies have either measured anxiety and depression in isolation or when measured concurrently, studies such as Holm‐Denoma et al. (2014) have reported dissociations between anxiety and depression symptoms, with significant relations between disordered eating and depression, but not anxiety. These findings are inconsistent with the broader literature in eating disorders where anxiety symptoms are commonly reported to arise prior to the onset of the eating disorder (Raney et al., 2008). Our results are therefore in line with the findings from clinical eating disorder samples.

In line with our hypotheses, there were no gender differences in disordered eating or depression symptoms in preadolescents. Our results contradict previous evidence from adolescents that has shown gender to be a moderator in the association between anxiety and disordered eating (O’Dea & Abraham, 1999). Regression models examining the interaction between gender and both anxiety and depression were also not statistically significant. These nonsignificant results are interesting in the context of pubertal, environmental and social changes that occur in adolescence, which may be key in explaining why gender is a moderator in this older sample, and it could be the case that these key factors have not yet emerged for the sample in this study. For example, Deardorff et al. (2007) reported an increase in social anxiety symptoms in females compared to males during puberty. This gender difference may be important in the context of disordered eating due to the overlap between eating disorders and social anxiety (Pallister & Waller, 2008).

The overlap between social anxiety and eating disorders is important when considering the mechanisms underlying the trajectories of anxiety and disordered eating. Pallister and Waller (2008) argue the relative chronology of anxiety and eating disorders is dependent on the type of anxiety. Social anxiety, for example, has been reported to frequently precede the eating disorder, while generalized anxiety disorder more commonly occurs later or simultaneously. This may be because social anxiety typically starts during childhood, whereas generalized anxiety disorder emerges during adulthood (Pallister & Waller, 2008). Schwalberg et al. (1992) suggest disordered eating behaviors and attitudes such as concerns over shape, eating, and weight may emerge as a result of anxiety about social evaluation; providing further support for a specific association between social anxiety and disordered eating and highlighting the role different types of anxiety may play in the etiology of eating disorders and disordered eating.

One of the aims of the study was to investigate levels of anxiety and depression symptoms among children who were categorized as above/below the clinical threshold for disordered eating. Based on the limited sample of children who were categorized as above the clinical threshold (*n* = 18), we were not adequately powered to test this.

### 4.1 Implications

This study has implications for broader understanding of the presentation of disordered eating and internalizing symptoms in preadolescence. Firstly, the presence of these potentially very serious behaviors and cognitions in a moderately large and representative sample of children is concerning and highlights the importance of early screening measures for prevention and intervention. In addition, the dissociation between anxiety and depression and their relations to disordered eating highlights the importance of measuring all three concurrently when examining the link between anxiety/depression and disordered eating in preadolescence.

Furthermore, the presence of disordered eating in both girls and boys supports early screening for disordered eating for all children; however, more targeted interventions could be better suited to adolescents due to the increased prevalence of eating disorders in females compared to males, indicating different risk trajectories.

The strong associations between disordered eating and anxiety in preadolescence during the end of primary school not only supports previous research in adolescence (Touchette et al., 2011; Zaider et al., 2000), but also contributes to the limited literature in preadolescence. Both disordered eating and anxiety during this stage of development have the potential to increase risk of developing eating disorders and anxiety disorders in later adolescence, and as highlighted already, early detection of disordered eating and anxiety may be important at this earlier stage. This is especially important considering the increased stress that can occur during this time as a result of the transition from primary to secondary school (Rice et al., 2011). A transdiagnostic prevention program during the last year of primary school would be one potential way of addressing this.

### 4.2 Limitations and future research ideas

One limitation of the methodology used in this study is the reliance on self‐report measures, and the biases that can occur when self‐report measures are used have been well‐documented (Paulhus, 1991). Importantly, the measures used in this study were adapted and validated questionnaires for the age group recruited in this sample, and previous research has demonstrated self‐report symptom scales are predictive of subsequent diagnoses (Shankman et al., 2009). In addition, children who required extra provision with comprehension and reading were provided with support by the researcher and/or schoolteacher during the testing sessions. Parent and teacher reports or diagnostic interviews with the children would have provided richer information; however, these methods are also constrained by time and resources.

The findings from our study were based on a cross‐sectional design so we are not able to examine the trajectories of disordered eating, depression, and anxiety across this period from preadolescence to adolescence or draw conclusions about causality. Therefore, longitudinal studies which start during preadolescence and follow‐up during adolescence would be valuable in examining the co‐occurrence of these symptoms.

Additionally, the use of opt‐in parental consent was important when recruiting for this study due to the sensitive nature of the questionnaires employed; however, there is potential for this to introduce sampling bias as some parents may be wary of research if there is little experience with the procedures, or if their child has presented with eating behaviors that are concerning, their parents may want to protect their child from engaging with the questionnaires at school.

Future research could also consider the measures used to examine disordered eating in preadolescence, as there are still inconsistencies present in the literature. Adjustments to the scoring method and factor structure of the ChEAT in nonclinical populations have been proposed to increase the variance of the item scores and hence the total score, as well as reducing the skewness in the data (Anton et al., 2006). In addition, future research could consider examining individual types of anxiety in this age group, such as social anxiety, as research suggests different types of anxiety could emerge at different ages and different stages of eating disorder development (Pallister & Waller, 2008).

## 5 CONCLUSION

This study provides support for the associations between disordered eating and both anxiety and depression in preadolescence. While there was evidence that the relation between anxiety and disordered eating behaviors is significant, and that this is the case for both boys and girls, depression was not a significant independent variable when included in the model alongside anxiety. These results highlight the importance of early detection for disordered eating behaviors and attitudes, as well as examining both anxiety and depression in boys and girls during preadolescence. Transdiagnostic interventions targeting several co‐occurring problems, such as disordered eating, anxiety, and depression might be effective for preventing the development of eating disorders in the long term. However, longitudinal research is vital to examine the trajectories of these problems, as well as additional factors, across time.

## CONFLICT OF INTEREST

We have no known conflict of interest to disclose.

## AUTHOR CONTRIBUTION

KT, MW, and RV were responsible for the conception and design of the project. KT led on participant recruitment and data collection. KT, MW, and RV all made substantial contributions to the analysis and interpretation of the data. KT led on drafting the manuscript, which MW and RV revised for important intellectual content. All authors viewed the manuscript and gave their approval before submission.

### Peer Review

The peer review history for this article is available at https://publons.com/publon/10.1002/brb3.1904.

## Supporting information

Table S1

## Data Availability

The data that support the findings of this study are openly available in Open Science Framework at https://osf.io/zpba4/, https://doi.org/10.17605/OSF.IO/ZPBA4 (Thomas et al., 2020).
