# Supplementary material for: Disordered eating and internalizing symptoms in preadolescence
Source: Brain Behav. 2020 Oct 19;11(1):e01904. doi: 10.1002/brb3.1904 (PMC7821606; doi:10.1002/brb3.1904)
Supplement: Supplementary file 1 — Table S1 [file BRB3-11-e01904-s001.docx]

**Supplementary Materials**

| **Table S1**  *Pearson’s Correlations Between Questionnaire Measures for Females* | | | | |
| --- | --- | --- | --- | --- |
|  | ChEAT | RCADS Total | RCADS Anxiety | RCADS Depression |
| ChEAT | 1 |  |  |  |
| RCADS Total | .442*** | 1 |  |  |
| RCADS Anxiety | .480*** | .953*** | 1 |  |
| RCADS Depression | .378*** | .883*** | .730*** | 1 |
| *Note.* ***Correlation is statistically significant at *p* < .001 | | | | |

| **Table S2**  *Pearson’s Correlations Between Questionnaire Measures for Males* | | | | |
| --- | --- | --- | --- | --- |
|  | ChEAT | RCADS Total | RCADS Anxiety | RCADS Depression |
| ChEAT | 1 |  |  |  |
| RCADS Total | .404** | 1 |  |  |
| RCADS Anxiety | .363** | .911** | 1 |  |
| RCADS Depression | .336** | .878** | .633** | 1 |
| *Note.* **Correlation is statistically significant at *p* < .01 | | | | |
